# Implementation of a Mobile Health Approach to a Long-Lasting Insecticidal Net Uptake Intervention for Malaria Prevention Among Pregnant Women in Tanzania: Process Evaluation of the Hati Salama (HASA) Randomized Controlled Trial Study

**DOI:** 10.2196/51527

**Published:** 2024-11-05

**Authors:** Trinity Vey, Eleonora Kinnicutt, Nicola West, Jessica Sleeth, Kenneth Bernard Nchimbi, Karen Yeates

**Affiliations:** 1 Department of Medicine Queen's University Kingston, ON Canada; 2 Management Sciences for Health Arlington, VA United States; 3 Pamoja Tunaweza Women’s Centre Moshi United Republic of Tanzania; 4 Tanzania Education Network/Mtandao wa Elimu Tanzania Dar es Salaam United Republic of Tanzania

**Keywords:** mHealth, short message service, behavior change communication, pregnancy, long-lasting insecticidal nets, malaria, protozoan infections, parasitic diseases, vector borne diseases, insecticide, intervention, malaria prevention

## Abstract

**Background:**

Malaria infection is associated with many adverse outcomes for pregnant women and neonates, yet pregnant women in East and Southern Africa remain frequently exposed to malaria. Long-lasting insecticidal nets (LLINs) can help prevent malarial infections and the associated adverse events. The Hati Salama (HASA) study was a cluster-randomized controlled trial implemented in 100 antenatal health facilities in urban and rural settings of Tanzania that provided pregnant women in both intervention and control groups with e-vouchers to redeem for LLINs for malaria prevention. The intervention group received behavior change communication mobile messages across a 14-day period while the e-voucher was active, and no significant difference between the rates of e-voucher redemption was found across the two groups.

**Objective:**

This study was a process evaluation of the HASA randomized controlled trial to determine barriers and facilitators to e-voucher reception and LLIN acquisition for pregnant women enrolled in the trial, as well as challenges and lessons learned by nurses who worked at the antenatal health facilities supporting the trial.

**Methods:**

Following the e-voucher’s expiration at 14 days, voluntary phone follow-up surveys were conducted for nurses who supported the trial, as well as participants in both intervention and control groups of the trial who did not redeem their e-vouchers. Survey questions asked nurses about workflow, training sessions, network connectivity, proxy phone use, and more. Surveys asked participants about reasons for not redeeming e-vouchers. Both surveys provided lists of preset answers to questions, as well as the option to provide open-ended responses. Nurses and trial participants were contacted between January and June 2016 on up to three occasions.

**Results:**

While nurses who supported the HASA trial seemed to recognize the value of the program in their communities, some barriers identified by nurses included network connectivity, workload increase, inadequate training and on-the-ground support, and difficulty following the workflow. Several barriers identified by trial participants included personal obligations preventing them from redeeming the e-voucher on time, network connectivity issues, losing the e-voucher number, no stock of LLINs at retailers when attended, inadequate explanation of where or how to redeem the e-voucher, or not receiving an SMS text message with the e-voucher number promptly or at all.

**Conclusions:**

Large-scale e-voucher platforms for health-related commodity interventions, such as LLIN distribution in sub-Saharan Africa, are feasible, but challenges, including network connectivity, must be addressed. Nurses identified issues to be considered in a future scale-up, such that the number of nurses trained should be increased and the e-voucher issuance workflow should be simplified. To address some of the key barriers impacting e-voucher redemption for trial participants, the network of retailers could be expanded and the e-voucher expiration period should be extended.

**Trial Registration:**

ClinicalTrials.gov NCT02561624; https://clinicaltrials.gov/ct2/show/NCT02561624

## Introduction

Malaria remains a significant cause of morbidity and mortality in sub-Saharan Africa [1]. Malaria infection in pregnant women is associated with a variety of adverse outcomes including miscarriage, neonate death, low birth weight, and maternal anemia and death [2]; yet in 2020, 22% of pregnant women in East and Southern Africa were exposed to malaria [1]. The use of insecticide-treated nets (ITNs) by pregnant women in malaria-endemic regions of Africa has shown decreased outcomes of placental malaria, low birth weight, and fetal loss [3]. As such, ITNs, and specifically, long-lasting insecticidal nets (LLINs), are a core malaria prevention tool, recommended by the World Health Organization [4].

In attempts to increase LLIN use in Tanzania, the Tanzania National Voucher Scheme (TNVS) provided pregnant women and women with young children with vouchers at antenatal clinics that could be redeemed for LLINs at a discounted price [5]. At the program’s inception in 2004, vouchers were distributed in paper format, until 2011 when electronic “e-vouchers” were implemented. Despite the high risk of maternal and neonate morbidity and mortality from malaria, findings suggest that only 47% of women who attended an antenatal clinic subsequently received a net through the TNVS [5].

Seeking to address this gap, the Hati Salama (HASA) study aimed to increase LLIN uptake in pregnant women [6]. The HASA study was a cluster-randomized controlled trial (RCT) in which pregnant women older than 13 years of age in Tanzania were issued e-vouchers through nurses at antenatal clinics that could subsequently be redeemed for LLINs at a subsidized price at a local retailer, where LLINs were stored and distributed. The original cost of an LLIN was approximately US $8, but at a retailer registered with the intervention, participants with an e-voucher only would pay approximately US $0.25 [6]. Throughout the 14-day period in which the e-voucher was active, the intervention group of women received behavior change communication SMS text messages emphasizing the importance of LLIN use during pregnancy and reminding them to redeem their e-vouchers. While there was no significant increase in LLIN uptake for the pregnant women in the intervention group compared to the control group that did not receive SMS text messages, with a total of 3746 e-vouchers redeemed, the HASA study demonstrated that LLIN e-voucher distribution via nurses in antenatal clinics is feasible on a large scale.

Findings from several studies using mobile health (mHealth) as a tool for malaria prevention and treatment have found that SMS text messages were well accepted by recipients [7-9]. However, there is a paucity of literature investigating the barriers to mobile messaging or e-voucher schemes from the perspective of both study recipients and administrators for malaria prevention.

This paper presents the mixed methods evaluation of the HASA study that was designed to assess perceived barriers to e-voucher redemption and LLIN acquisition through follow-up surveys administered to nurses and study participants of the HASA study. In doing so, we highlight challenges, unintended findings, and lessons learned to ensure effective scale-up and implementation of the e-voucher platform for future health-related commodity interventions such as LLIN distribution in sub-Saharan Africa.

## Methods

### The HASA RCT

The HASA study was a cluster-randomized controlled double-blind 2-arm study implemented between July and December 2015, in 100 antenatal health facilities in urban and rural settings across 21 regions of Tanzania (ClinicalTrials.gov ID NCT02561624) [6]. There were 50 clusters randomly allocated to the intervention and 50 clusters to the control group. Participants were pregnant women older than 13 years of age attending one of the selected antenatal health facilities, who were either literate or living with someone who could assist them with reading SMS text messages. This research study presents the mixed methods evaluation of the implementation of the HASA cluster RCT. This mixed methods evaluation took place via nurse and participant surveys, described in more detail below, between January and June 2016.

### Staff Training

Prior to the implementation of the HASA RCT, centralized meetings were organized with all relevant medical stakeholders from enrolled antenatal clinics including Regional Medical Officers, District Medical Officers, and Clinic Officers-in-Charge. The objectives of these meetings, led by one representative from Queen’s University and one from Mennonite Economic Development Associates (MEDA) Tanzania, were to present the HASA trial, answer any questions or concerns, and define the stakeholder’s involvement during the implementation and follow-up period of the trial. The meetings also provided an opportunity to obtain informed consent from all medical and clinic officers agreeing to have their health facility participate in the study and adhere to all ethical procedures of the trial.

Medical officers from each enrolled health facility selected two nurses from the facility’s antenatal department for training and participation in the HASA trial. Three nurse training sessions and three retailer training sessions were held between June 1 and June 8, 2015, in the Tanzanian cities of Mwanza, Dodoma, and Dar es Salaam. Sessions were attended by more than 180 nurses from the 100 health facilities selected for the study, along with 28 retailers. For facility sites for which only one nurse could attend the training, this nurse subsequently trained the second nurse involved at their site. The objectives of the nurse training were to introduce the HASA trial, explain and review the role and participation of nurses in the trial, provide training regarding obtaining informed consent and standard operating procedures, and provide an overview of the SMS text messaging workflow. The objectives of the retailer training were to provide a thorough participatory overview of the retailer contract and an overview of the SMS text messaging workflow.

The registered retailers received small incentives for participating in the study; specifically, they received a percentage of the profits for LLINs sold through the program, they received increased foot traffic from women enrolled in the program, and at the end of the program, the retailers were able to sell the unredeemed LLINs for their profit. The retailers involved in the e-voucher program were all involved in the program managed by MEDA which predated the launch of the e-voucher program in the HASA study [6]. The retailers were small to medium-sized local convenience shops known in Tanzania as “Dukas.” To be included in the e-voucher program, they were within a 500-m radius of the catchment area of the health facility that was participating in the distribution of the e-vouchers.

Four zonal managers were selected from a pool of highly qualified former TNVS staff recommended by MEDA Tanzania to oversee the fieldwork. Each zonal manager was allotted a cluster of regions (zones) for which they were responsible for registering retailers and nurses prior to study implementation, as well as monitoring and managing the e-voucher workflow (both with regards to e-voucher issuance, as well as LLIN redemption at the retailer) to provide sufficient support, technical assistance, and retraining throughout the course of the study. Zonal managers were also responsible for data collection via iForm and providing weekly reports from facilities regarding women enrolled, vouchers issued, and supply of nets to retailers.

### Nurse Follow-Up Survey

Following the e-voucher expiration period of 14 days, a structured follow-up survey was conducted for nurses who worked at the antenatal health facilities enrolled in the trial. The survey was conducted directly over the phone by the HASA field team members. Nurses were contacted on up to 3 occasions with the option to participate in the survey. Nurses were asked a series of yes or no questions in addition to 2 open-ended questions (4b and 11), as listed in Table S1 in Multimedia Appendix 1. Responses were deidentified and uploaded into an iForm. A total of 180 antenatal nurses were involved in the HASA study and contacted to be asked to participate in this phone survey, 67 of which did participate. Nurses from across the 21 regions of Tanzania involved in the study were contacted.

### Participant Follow-Up Survey

Following the e-voucher expiration period, all participants (in both intervention and control groups) who did not redeem their e-vouchers for an LLIN at a registered retailer were contacted by a HASA field team member by phone on up to 3 occasions to complete a voluntary structured follow-up survey over the phone. The survey asked participants to indicate the reason they did not redeem the e-voucher prior to its expiration. The survey included a list of preset options for participants to choose from, along with the option to provide an open-ended response for why the voucher was not redeemed, as listed in Table S2 in Multimedia Appendix 1. Responses were deidentified and uploaded into an iForm. Of the 1702 participants of the HASA trial from across 21 regions of Tanzania who did not redeem their e-vouchers and were thus contacted to be asked to participate in this phone survey, 410 participants did participate.

### Ethical Considerations

This study was conducted as a follow-up process evaluation under the HASA study, which received ethical approval from the National Institute of Medical Research Tanzania and Queen’s University Health Sciences and Affiliated Teaching Hospitals Research Ethics Board (6013000). Ethical approval to assess the feasibility, acceptability, and adoptability of the HASA intervention among nurses and women who participated in the intervention via surveys was obtained under the approval by the National Institute of Medical Research and Health Sciences (original certificate number NIMR/HQ/R.8a/Vol. IX/1750) and Affiliated Teaching Hospitals Research Ethics Board for the initial HASA RCT study. Phone surveys administered to both nurses and intervention participants began with a statement about their purpose and informed consent. Verbal consent was obtained, and confidentiality and anonymity were explained to participants.

## Results

Of the 180 antenatal clinic nurses involved in the study, 67 nurses provided answers to the phone survey following the e-voucher redemption period, which are listed in Table 1. The majority responded affirmatively that they felt HASA was helpful in improving the overall community understanding of malaria (n=65, 97%), and that women were eager and easily able to redeem for a net (n=65, 97%). All nurses surveyed (n=67, 100%) felt that their community needs programs like HASA. Only 67% (n=45) of nurses felt that the workflow was easy to follow, and only 37% (n=25) of nurses felt that the training and support provided were adequate. The majority (n=62, 93%) of nurses identified that they did experience challenges regarding network and connectivity, of which 92% (n=56) of nurses felt these issues impacted the number of vouchers issued or redeemed. While 91% (n=61) of nurses felt that using a proxy phone was helpful, just over half (n=35, 52%) of nurses experienced challenges finding a reliable proxy phone. A total of 73% (n=49) of nurses felt that involvement in HASA heavily increased their workload, and most (n=64, 96%) nurses agreed that having more nurses in their facility trained as issuers would have been helpful.

Responses to the open-ended question to nurses “If (you did not find that the workflow [to issue vouchers] was easy to understand and follow), what would you change?” (4b from Table S1 in Multimedia Appendix 1) are listed in Table 2. These answers were all centered around shortening and simplifying either the workflow or the SMS text messages.

Responses to the open-ended question “In a potential scale-up, what are your recommendations for improving the program?” (11 from Table S1 in Multimedia Appendix 1), which were suggested by nurse respondents in the survey are listed in Table 3. Nurses were able to provide up to three suggestions each. Many responses suggested that network connectivity should be improved (n=38, 28.5%), more nurses should be trained to issue vouchers (n=27, 20%), and workflow should be shortened (n=10, 7.5%).

In total, of the 1702 participants who participated in the trial and did not redeem their e-vouchers, 410 participants completed the phone survey following the e-voucher redemption period, indicating the reason they did not redeem the e-voucher [6]. The answers to this survey are listed in Table 4. Common reasons that participants did not redeem their voucher included that they lost their voucher number (n=64, 16%), they went to the shop, but the shopkeeper did not use the e-voucher system (n=54, 13%), or they went to redeem the e-voucher, but the store had no nets available (n=23, 6%). Over half (n=217, 53%) of participants who responded to the survey indicated that there was an “other reason” they did not redeem the e-voucher.

The open-ended responses detailing other reasons that participants did not redeem their e-voucher for an LLIN are listed in Table 5. Participants were able to provide multiple reasons. There is some overlap between the responses detailed in Table 5 and those in the initial participant survey (Table 4), however, most of the open-ended responses were distinct from the initial survey. The most common of these was that participants could not redeem their e-voucher on time due to a variety of other obligations or health concerns (n=90, 35%), participants did not receive an SMS text message with the e-voucher at all or on time (n=38, 14.8%), and participants did not receive an adequate explanation of the HASA program and e-voucher redemption (n=26, 10.1%).

**Table 1:** Responses to the nurse follow-up survey.

Questions and responses		Count	Percentage
**1. Did you find HASA^a^ to be helpful in improving the overall perception and understanding of malaria and the importance of prevention? Both with regards to the bed net component as well as messages?**			
	Yes	65	97
	No	2	3
**2. Do you find that this is a program that your community needs?**			
	Yes	67	100
	No	0	0
**3. Do you find that your patients were eager to and easily able to redeem a net?**			
	Yes	65	97
	No	2	3
**4. Do you find that the workflow (to issue vouchers) was easy to understand and follow?**			
	Yes	45	67
	No	22	33
**5. Did you find the training sessions and on-the-ground support adequate?**			
	Yes	25	37
	No	42	63
**6a. Did you encounter challenges with regard to network or connectivity when issuing vouchers?**			
	Yes	62	93
	No	5	7
**6b. If so, did this affect the number of vouchers you issued or redemption by beneficiaries?**			
	Yes	56	92
	No	5	8
**7. Considering your catchment population, was it challenging to register women with phones?**			
	Yes	40	60
	No	27	40
**8a. Did you find that using a proxy phone was helpful?**			
	Yes	61	91
	No	6	9
**8b. Did you encounter challenges with finding a reliable proxy phone?**			
	Yes	35	52
	No	32	48
**9. Did you find that your involvement in HASA increased your workload heavily?**			
	Yes	49	73
	No	18	27
**10. Do you think it would have been helpful to have had more nurses trained as issuers in your facility?**			
	Yes	64	96
	No	3	4

^a^HASA: Hati Salama.

**Table 2:** Responses from nurse survey to open-ended question 4b “If you did not find that the workflow was easy to understand and follow, what would you change?”

Response	Count (n=14)	Percentage
Workflow should be shortened and simplified	10	72
Only one SMS text message should be sent	3	21
SMS text messages should be shortened	1	7

**Table 3:** Responses from nurse survey to open-ended question 11 “In a potential scale-up, what are your recommendations for improving the program?”

Responses	Count (n=134)	Percentage
Network connectivity should be improved	38	28.5
More nurses or clinic staff should be trained to issue vouchers	27	20
Workflow should be shortened	10	7.5
LLINs^a^ should be available within, or closer to, the health facility	8	6
An alternative way to issue vouchers to participants without phones should be considered	7	5
More training sessions for voucher issuers is needed	6	4.5
Staff bonus or motivation for voucher issuers should be considered and given on time	6	4.5
The health care facility or program should provide a phone that can be used for voucher issuance	6	4.5
The number of forms that voucher issuers need to fill out should be reduced	5	4
Nurses should receive a message with the voucher number to confirm that clients have received the voucher	4	3
The retailer shops should have a greater stock of LLINs	4	3
LLINs should also be issued to children younger than 5 years	3	2
Women without phones should continue to use a proxy phone	2	1.5
There should be more than one retailer associated with each health facility	2	1.5
LLINs and vouchers should be issued at regular mobile clinics	1	0.75
One SMS text message during issuance is enough	1	0.75
There should be posters that explain the program	1	0.75
LLINs of different sizes and colors should be available	1	0.75
LLIN mesh size should be smaller	1	0.75
Vouchers should be available more promptly	1	0.75

^a^LLIN: long-lasting insecticidal net.

**Table 4:** Participant follow-up survey responses.

	Responses	Count (n=410)	Percentage
1	I lost my voucher number	64	16
2	My voucher number was not valid when I took it to the shop	9	2
3	I went to redeem the voucher and the store had no nets available	23	6
4	I do not want a net	10	2
5	I have all the bed nets I need	3	1
6	I cannot afford to pay the additional payment for the net	12	3
7	I do not live close enough to the shops that are in the Hati Punguzo program	18	4
8	I went to the shop, but the shopkeeper failed to use the e-voucher system	54	13
9	Other reason	217	53

**Table 5:** Answers to participant follow-up survey provided for “other reason.”

Responses	Count (n=257)	Percentage
Participant was not able to redeem e-voucher prior to expiration due to illness, giving birth, travel, or other obligations	90	35
Participant never received an SMS text message with the e-voucher number, or the SMS text message did not arrive in time to redeem	38	14.8
Participant did not receive an adequate explanation about how or where to redeem the e-voucher	26	10.1
Issue related to proxy phone use (such as the owner of the phone was traveling)	18	7
Retailer shop was closed when the participant tried to redeem	16	6.2
The phone was lost, stolen, or damaged	12	4.7
No particular reason indicated	9	3.5
Forgot to redeem or negligence	8	3.1
Network connectivity issues at issuance or redemption of the e-voucher	7	2.7
Retailer shops are too far from where the participant lives	6	2.3
No instruction was given at the retail shop or the retailer was not informed about HASA^a^	5	1.9
The SMS text message was deleted from the phone	5	1.9
The husband did not understand the HASA program	3	1.2
Confusion about the expiry date or unaware it expired	3	1.2
LLIN^b^ stock issue	2	0.8
Participant already had sufficient LLINs	2	0.8
Did not like the retailer’s attitude	2	0.8
The SMS text message was sent to the wrong number	2	0.8
Did not want white-colored LLIN	1	0.4
LLIN described as too stiff	1	0.4
Participant did not bring antenatal clinic card to the retailer staff and they would not sell them the LLIN	1	0.4

^a^HASA: Hati Salama.

^b^LLIN: long-lasting insecticidal net.

## Discussion

### Principal Results

This process evaluation study sought to understand the challenges experienced during the HASA trial by both participants who received e-vouchers to redeem for LLINs and the nurses at antenatal clinics who issued these vouchers.

The responses to the nurse follow-up survey indicated that nurses recognized the value of the HASA program, with 97% (n=65) of nurses responding that it was helpful in improving the overall perception and understanding of malaria and the importance of prevention. Similarly, 100% (n=67) of nurses surveyed felt that HASA is a program that their community needs. This positive reception of the program supports the finding from the HASA primary outcome paper that nurses likely played a role in motivating participants and encouraging LLIN uptake in both control and intervention groups [6]. This also suggests that such interventions surrounding LLIN use in pregnant women will be well-received and supported by community nurses.

The nurse survey did identify several key process barriers, with 33% (n=22) of nurses responding that the workflow was not easy to follow, and 63% (n=42) of nurses responding that the training sessions and on-the-ground support were not adequate. These findings were emphasized during the open-ended question responses, with suggestions focused on shortening workflow (n=10, 7.5%), reducing the number of forms for nurses to fill out (n=5, 4%), and providing more training sessions (n=6, 4.5%). Together, this suggests that the workflow and training for staff should be revised and simplified and that perhaps zonal managers should be empowered to provide greater on-demand support and retraining. It is also possible that since 33% (n=22) of nurses found the workflow not easy to follow, this may have impacted their explanations of the program to participants and subsequent redemption; 10% (n=26) of participants did not redeem their e-voucher due to feeling that they did not receive an adequate explanation of the program. As such, simplifying workflow in the future may not only improve nurse satisfaction with the program but may increase reception.

A total of 73% (n=49) of nurses surveyed felt that HASA increased their workload heavily, with 96% (n=64) of nurses agreeing that having more nurses trained as issuers would have been helpful. During the implementation stage of the HASA trial, the ongoing absence of nurses from their postings due to conflicting obligations (work seminars and commitments, family obligations, illness, etc) was noted by the HASA field staff. Such understaffing of voucher issuers likely contributed to many nurses feeling that their workload was increased. Together, this demonstrates the need to train more staff to issue vouchers, including junior nurses with lower levels of additional responsibilities, who will often be present in the facility. Providing a staff bonus or additional incentive, as recommended by 4.5% (n=6) of respondents, may also help decrease the feeling of being overworked.

### Comparison With Prior Work

An additional recommendation by 6% (n=8) of nurses was that LLINs should be sold within the antenatal clinic itself. This is consistent with suggestions from clinic staff in a similar Tanzania ITN study, who felt that the purchase of discounted nets should only take place at the clinic [10]. While this may improve redemption, an overall strength of the HASA study was that the retailers were generally in close proximity to the antenatal clinics, allowing some women to redeem vouchers immediately following their visit. The geographical accessibility of the retailers did not seem to present an especially large challenge to redemption for participants. Some nurses also suggested that only one SMS text message, or shortened messages, should be sent to participants. This is reflective of the literature on mHealth interventions in Ghana and Kenya showing that a short simple message can be as effective as sending longer or multiple messages and therefore could be considered for the scale-up of such a mHealth initiative [11,12].

A major challenge identified by both nurses and participants was issues surrounding network and connectivity. A total of 93% (n=62) of nurses encountered network challenges, the majority of which impacted voucher issuance or redemption. It is possible that network issues may be amplified for women of lower socioeconomic status, who may be more likely to live in an area with unreliable cellular connectivity. Women of lower socioeconomic status have already been found to have lower ITN voucher uptake compared to those of higher socioeconomic status [13,14]. This reflects the most common suggestion in the literature from participants of an ITN voucher scheme in Tanzania that the monetary value of vouchers should be increased [10]. In rural areas, which are typically already subject to unreliable connectivity, research suggests that women are less likely to be the sole owners of phones, which may cause additional barriers through the use of proxy phones [15]. In a future scale-up, network reliability may present less of an issue, as cellular coverage continues to increase in rural areas of Tanzania and sub-Saharan Africa. A technical backup system could also be implemented in the future to avoid issues with e-voucher issuance or redemption due to network connectivity.

One barrier to nonredemption identified by 6% (n=23) of participants was that the retailer had no nets available when they went to redeem the voucher. This supports a similar recommendation from the nurse survey that retailer shops should have a greater stock of LLINs. For the HASA study, the stock of LLINs was delivered to local retailers at multiple phases during the program with numbers of LLINs provided based on historical data and the number of participants each site was expected to enroll. Despite retailers signing a contract that the LLINs were intended to be exclusively used for the HASA program, some retailers may have sold stock to other customers. Particularly since part of the incentive for retailers to participate in the intervention was that they were able to sell unredeemed LLINs following the trial for profit, it is possible that retailers intentionally or unintentionally sold LLINs outside the trial before it ended, thus impacting stock. Concerns around fraudulent e-voucher creation by retailers’ staff to sell LLINs on the commercial market were in part addressed by an e-voucher redesign; security for e-vouchers was increased by including a unique identifier number for all pregnant women, random barcode activation at the retailer, and reports to verify the number of e-vouchers issued by clinics. A similar voucher scheme for ITNs in Ghana also found that inadequate stock of ITNs was the greatest issue faced by retailers [16]. An additional barrier encountered at retailers was that 13% (n=54) of participants attended a shop to redeem their e-vouchers, but the shopkeeper did not recognize the e-voucher system. This could potentially be overcome in a scale-up by expanding the network of retailers using the e-voucher system.

While a Ghana voucher scheme found that the major reason (36%) for women not accepting a voucher at an antenatal clinic was that they already had a net, this was a minor reason for nonredemption in the HASA study [16]. Only 1% (n=3) of women stated that they already had all the nets they needed, suggesting that despite the need for LLINs, there were other barriers preventing the redemption of e-vouchers. Another reason identified preventing redemption for 10% (n=26) of participants was that they did not receive a sufficient explanation about how to redeem the voucher. This is consistent with suggestions from participants of another ITN voucher scheme in Tanzania that more information about the voucher scheme, malaria, and nets is required [10]. Another similar study suggests that a barrier to receiving vouchers at antenatal clinics was that clinic coordinators were not familiar enough with the voucher system [17]. More thorough training and regular retraining of nurses to ensure they are providing adequate explanations of the e-voucher scheme to women may alleviate this barrier. A total of 16% (n=64) of women indicated that they lost their voucher number, which may have been due to proxy phone use if another user of the phone deleted the SMS text message with the voucher code. It is also possible that some participants could not find the SMS text message on their phone at the time they went to redeem it, or that the message may have been deleted in error.

The main reason provided that participants did not redeem their e-voucher (n=90, 35%) reflected that the voucher expired before the participant had a chance to redeem it; this barrier could be resolved by extending the expiration period beyond 14 days. In 2011, prior to the switch from paper vouchers to e-vouchers, the TNVS vouchers had a validity of 60 days [5]. Extending the e-voucher validity period or giving participants the option to reregister if the deadline is missed may improve redemption.

### Limitations

A potential limitation of this study was that surveys of nurses and participants were completed over the phone. While this decreased costs and travel time associated with in-person interviews, potentially facilitating responses from rural participants, this method likely limited the responses from those without access to their phones. Despite the frequency of yes and no questions and preset answers, nurses and participants were given the opportunity to provide their open-ended recommendations, enriching the data collected. Due to the survey format, there was an element of interpretation required for the open-ended responses given by both nurses and participants. Despite providing up to 3 opportunities for both nurses and intervention participants to participate in these surveys, response rates to the surveys were 67 of 180 nurses and 410 of 1702 study participants, which suggests that there are likely challenges and facilitators of the HASA study experienced by nurses and participants that are not captured in this paper.

### Conclusions

Despite these identified challenges, the HASA study was a large robust trial with an overall positive perception by involved nurses, suggesting that large e-voucher platforms for health-related commodity interventions, such as LLIN distribution in sub-Saharan Africa, are feasible [6]. In a scale-up of HASA or similar programs, many of the issues identified by nurses could be overcome by facilitating the nurse workflow and training a greater number of nurses. To address some of the major barriers faced by participants, we suggest extending the validity of the e-voucher and expanding the network of retailers selling LLINs for the program. Improving network connectivity would facilitate the HASA program for both nurses and participants.

## Appendix

Multimedia Appendix 1 Additional tables.

## Abbreviations

HASA: Hati Salama
ITN: insecticide-treated net
LLIN: long-lasting insecticidal net
MEDA: Mennonite Economic Development Associates
mHealth: mobile health
RCT: randomized controlled trial
TNVS: Tanzania National Voucher Scheme

## Data Availability

The datasets generated during or analyzed during this study are available from the corresponding author upon reasonable request.
