# Supplementary material for: Implementation of a Mobile Health Approach to a Long-Lasting Insecticidal Net Uptake Intervention for Malaria Prevention Among Pregnant Women in Tanzania: Process Evaluation of the Hati Salama (HASA) Randomized Controlled Trial Study
Source: J Med Internet Res. 2024 Nov 5;26:e51527. doi: 10.2196/51527 (PMC11576607; doi:10.2196/51527)
Supplement: Multimedia Appendix 1 [file jmir_v26i1e51527_app1.docx]

**Table S1.** Nurse follow-up survey questions (translated from Swahili to English).

|  | Preset question content |
| --- | --- |
| 1 | Did you find HASA^a^ to be helpful in improving the overall perception and understanding of malaria and the importance of prevention? Both with regards to the bed net component, as well as messages? |
| 2 | Do you find that this is a program that your community needs? |
| 3 | Do you find that your patients were eager to and easily able to redeem a net? |
| 4a | Do you find that the workflow (to issue vouchers) was easy to understand and follow? |
| 4b | If not, what would you change? |
| 5 | Did you find the training sessions and on-the-ground support adequate? |
| 6a | Did you encounter challenges with regard to network or connectivity when issuing vouchers? |
| 6b | If so, did this affect the number of vouchers you issued or redemption by beneficiaries? |
| 7 | Considering your catchment population, was it challenging to register women with phones? |
| 8a | Did you find that using a proxy phone was helpful? |
| 8b | Did you encounter challenges with finding a reliable proxy phone? |
| 9 | Did you find that your involvement in HASA increased your workload heavily? |
| 10 | Do you think it would have been helpful to have had more nurses trained as issuers in your facility? |
| 11 | In a potential scale-up, what are your recommendations for improving the program? |

^a^HASA: Hati Salama.

**Table S2.** Participant follow-up survey answer options (translated from Swahili to English).

|  | Preset answer content |
| --- | --- |
| 1 | I lost my voucher number |
| 2 | My voucher number was not valid when I took it to the shop |
| 3 | I went to redeem the voucher and the store had no nets available |
| 4 | I do not want a net |
| 5 | I have all the bed nets I need |
| 6 | I cannot afford to pay the additional payment for the net |
| 7 | I do not live close enough to the shops that are in the Hati Punguzo program |
| 8 | I went to the shop, but the shopkeeper failed to use the e-voucher system |
| 9 | Other reason (open-ended response) |
